# Case Report: When an unknown fever unmasks Erdheim-Chester disease: a case showing the use of multimodality imaging and the successful interferon alpha therapy.

**DOI:** 10.12688/f1000research.169202.2

**Published:** 2025-10-17

**Authors:** Hajer Boudriga, Imen Ben Hassine, Omar Ben Elkilani, Raja Sfar, Abir Ezzine, Jihed Anoun, Anis Mzabi, Fatma Ben Fredj, Kaouthar Chatti

**Affiliations:** 1 Department of internal medicine-Sahloul University Hospital, University of Sousse, Sousse, Tunisia; 2 Department of Nuclear Medicine-Sahloul Universty Hospital, University of Sousse, Sousse, Tunisia

**Keywords:** Erdheim-Chester disease; histiocytosis, non-langerhans-cell; diabetes insipidus, neurogenic; Positron-Emission Tomography; diagnostic imaging; interferon-alpha

## Abstract

**Background:**

Erdheim-Chester disease (ECD) is an extremely rare histiocytic neoplasm with a highly variable and often unpredictable clinical course and prognosis. Multiple organs can be affected mimicking other systemic diseases both clinically and radiologically. This makes timely diagnosis more challenging.

**Case Presentation:**

We report the case of a 39-year-old male presented with a 15-day history of fever and retrosternal chest pain. Ha has a long history of diabetes insipidus, hypogonadism of unknown aetiology. On exploration, computed tomography revealed retroperitoneal fibrosis and “hairy” kidney appearance. Subsequent multimodality imaging studies showed the extensive multisystemic involvement including the skeletal system, retroperitoneum, pituitary, retroperitoneum, kidneys, heart, central nervous system, myocardium, pleura, lymph nodes and GI tract. Histopathology confirmed CD68+/CD1a- histiocytosis. Molecular analysis for BRAF mutation is currently pending. Pegylated interferon α and corticosteroids induced rapid clinical and biochemical improvement. Therapeutic response using a follow-up 18F-FDG PET scan revealed subsequent improved systemic lesions.

**Conclusion:**

A prolonged history of unexplained endocrine abnormalities in the context of fever of unknown origin should evoke the diagnosis of ECD. Multimodal imaging is essential for diagnosis and for monitoring treatment response. This case highlights the importance of the multidisciplinary approach that integrates clinical evaluation, histological analysis, and imaging studies for the optimal the management of this disease. Early initiation of immunomodulatory therapy can lead to better outcomes, even prior to confirmation of molecular findings.

## Introduction

Erdheim-Chester Disease (ECD) is an extremely rare haematological malignancy, characterized by the abnormal proliferation and deposition of histiocytes thus classified among the non-Langerhans-cell histiocytosis. The onset of ailment is typically in the middle age. Biopsy is the key element for the diagnosis through the integration of xanthogranuloma histology with compatible clinical and radiographic findings with the support of molecular analysis. ^1^

The disease can produce a variety of symptoms depending on the sites affected by the tumoral cell proliferation with the possibility of having multiple organs involved. ^2, 3^ Long bones involvement is nearly universal and is a key element of positive diagnosis. Extraskeletal involvement is seen in nearly half of the cases ^4^ and includes potentially life-threatening or disabling manifestations such as central nervous system infiltration, recurrent pericardial or pleural effusion, hypopituitarism, retroperitoneal fibrosis, and exophthalmos. ^2, 5^ The diagnostic process of ECD is often complex and based on biopsy, and increasingly molecular testing. Imaging, nevertheless, has a central role in guiding the diagnostic process, both in lesion detection and biopsy planning. Functional imaging, such as Fluorine-18 fluorodeoxyglucose positron emission tomography/computed tomography 18F-FDG PET/CT is especially useful in identifying multisystem involvement and monitoring therapeutic response. ^1, 4, 6^

We discuss in this case report of a patient with ECD, the impact of multimodality imaging on the diagnostic workup and therapeutic management of this rare malignancy.

## Case report

A 39-year-old man presented with a 15-day history of persistent fever peaking at 39-39.5 °C, associated with retrosternal chest pain radiating to the shoulders. He reported dull, bilateral ankle pain which had preceded the fever by several weeks with no associated swelling or erythema or joint stiffness. His medical history consisted of central diabetes insipidus diagnosed a year before the current admission and was managed with desmopressin, and secondary hypogonadism under endocrinologic follow-up. There was no significant family or psychological history.

On physical examination, the patient was febrile but haemodynamically stable. There were no overt signs of arthritis or joint inflammation. Neurological examination was normal, and a right sided exophthalmos was noted. No cutaneous abnormalities were identified.

Laboratory evaluation revealed elevated inflammatory markers, including a CRP level of 229 mg/L, and a mild thrombocytosis, raising initial concern for an underlying infectious, inflammatory, or myeloproliferative process.

Infectious causes were ruled out during primary investigations with negative viral serologies, unremarkable blood cultures and sterile urine testing.

**Figure 1.**
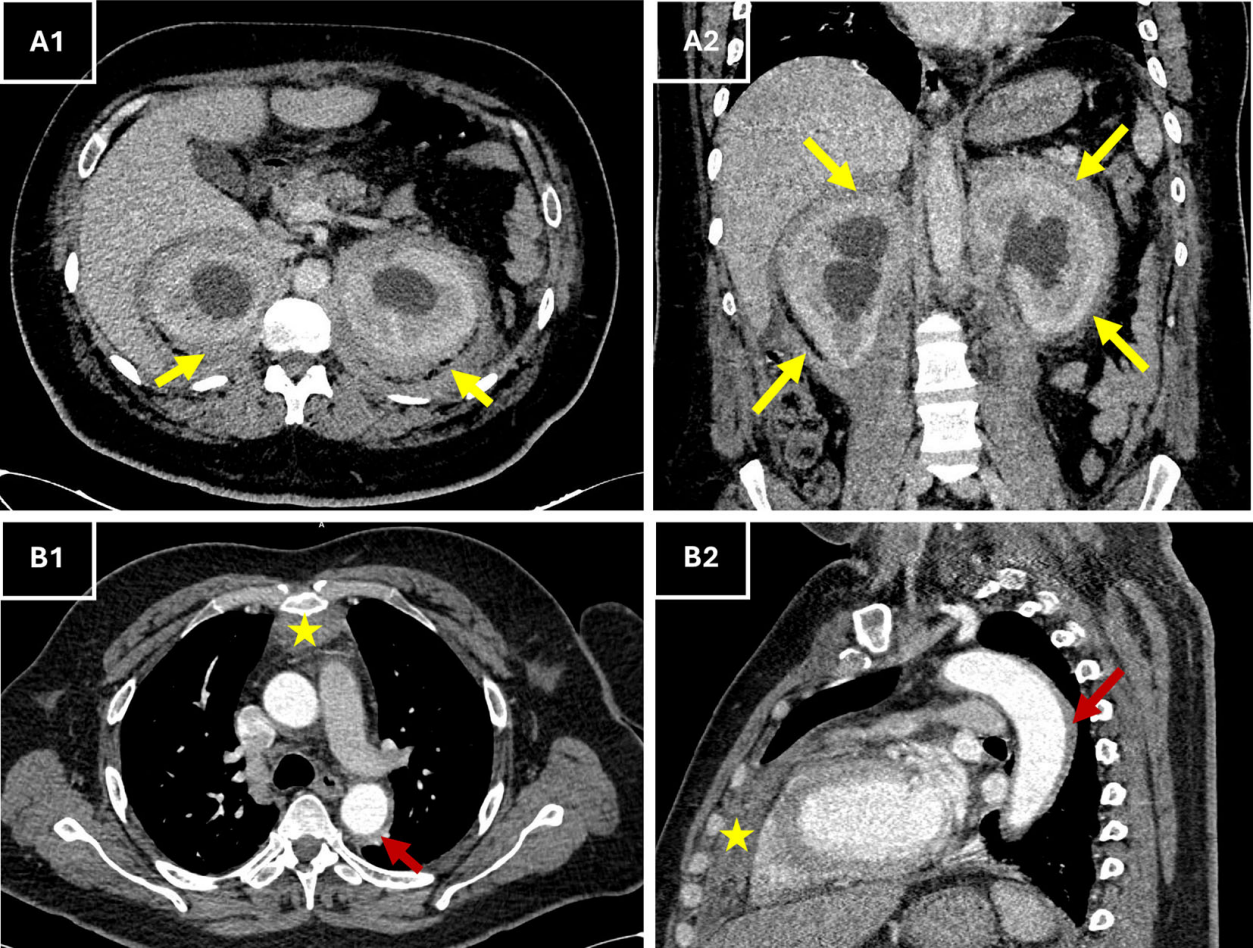
Contrast-enhanced CT images showing characteristic abdominal and thoracic involvement in ECD. (A1–A2) Axial (A1) and coronal (A2) contrast-enhanced CT views showing circumferential, bilateral perirenal soft tissue thickening (yellow arrows) consistent with the “hairy kidney” sign. (B1–B2) Axial (B1) and sagittal (B2) CT views showing retrosternal anterior mediastinal soft tissue thickening (*, red arrows) extending to the pericardium, in keeping with pericardial and mediastinal involvement described on the report. **Abbreviations**: **CT** = computed tomography; **ECD** = Erdheim–Chester disease.

In the context of acute chest pain and fever, differential diagnoses included aortic dissection, infective endocarditis, pericarditis, and vasculitis. Urgent thoracic computed tomography (CT) angiography excluded vascular emergencies and instead revealed a perivascular fibrosis of the descending aorta, a moderate pericardial effusion, anterior mediastinal fat infiltration along with symmetric retroperitoneal infiltration that surrounded the kidneys and extending to proximal ureters. This “hairy kidney” appearance raised suspicion of Erdheim-Chester disease ( Figure 1).

**Figure 2.**
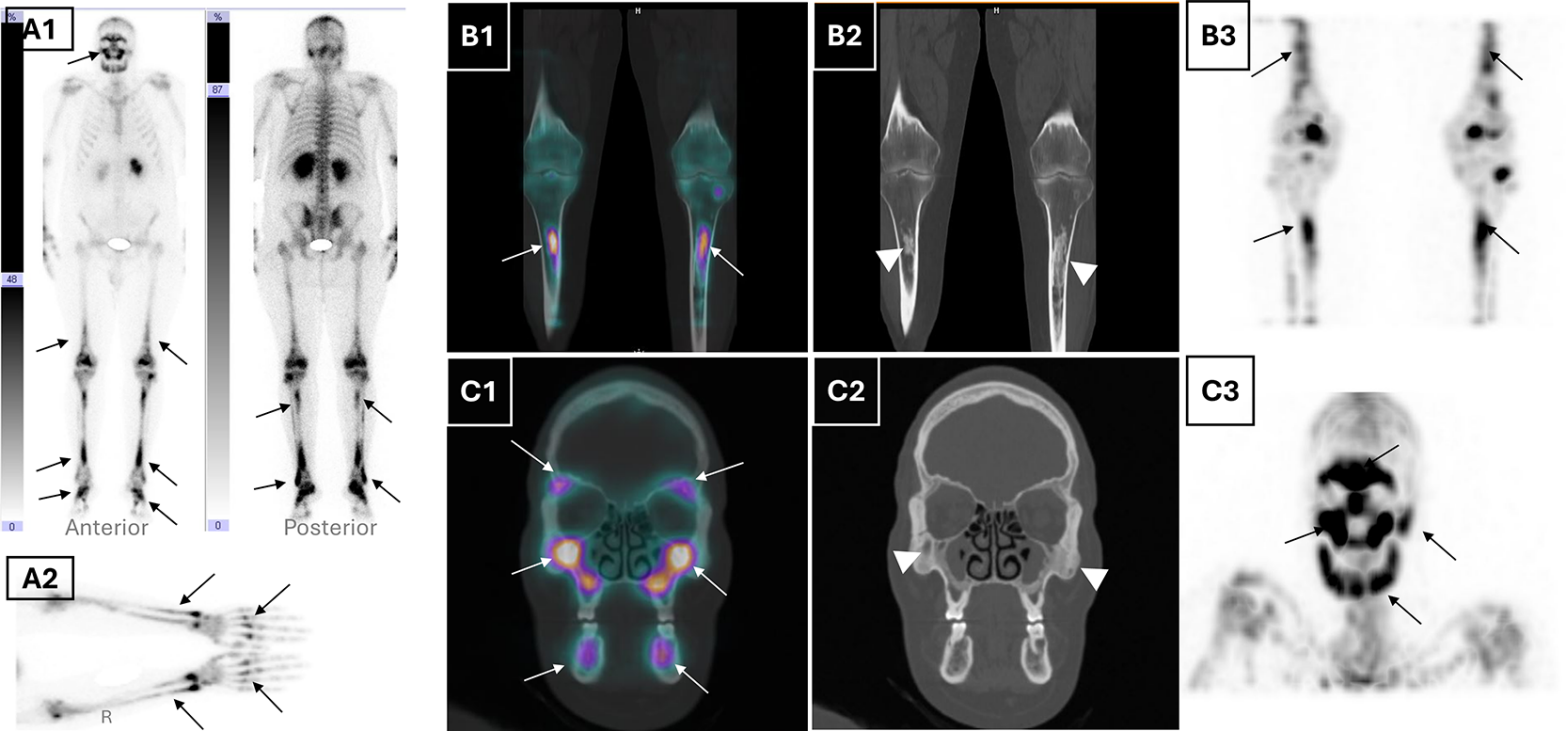
Multimodal nuclear bone imaging demonstrating typical skeletal and craniofacial involvement in ECD. (A1) Whole-body planar bone scintigraphy (anterior and posterior views) shows diffuse symmetric and bilateral metaphyseal-diaphyseal increased radiotracer uptake in the distal femora, tibiae, feet, and maxillofacial skeleton (black arrows), consistent with systemic osseous involvement. (B1) Coronal fused SPECT/CT and (B2) corresponding CT of the lower limbs show bilateral metaphyseal-diaphyseal uptake in the femurs and tibiae (white arrows), with associated cortical thickening and sclerosis (white arrowheads). (A2) Static planar view of the hands and forearms highlights pronounced tracer uptake in the metacarpals and distal radius/ulna (black arrows), supporting upper extremity involvement, which is less commonly reported in Erdheim-Chester disease. (B3) Coronal MIP SPECT image confirms symmetrical uptake in the long bones of the lower extremities (black arrows). (C1) Coronal fused SPECT/CT and (C2) CT of the maxillofacial bones highlight tracer accumulation in the maxillae, zygomatic arches, and mandibles (white arrows) and corresponding sclerotic changes (white arrowheads). (C3) MIP SPECT image of the skull reveals diffuse symmetric craniofacial tracer uptake, notably involving the orbitomaxillary complex and mandible (black arrows). **Abbreviations**: **SPECT** = single photon emission computed tomography; **CT** = computed tomography; **MIP** = maximum intensity projection; **ECD** = Erdheim–Chester disease.

Given the forementioned pathognomonic sign, and to further investigate skeletal involvement, a whole body 99mTc-HDP bone scintigraphy with single photon computed tomography/computed tomography (SPECT/CT) was performed. It demonstrated a markedly increased and symmetric radiotracer uptake in the lower femoral extremities, tibial bones, feet, and forearms, consistent with long bone osteosclerosis ( Figure 2). Additionally, tracer uptake was noted in the sphenoid, maxillary, and frontal sinuses with cortical thickening of the mandible, along with bilateral retro-orbital tracer accumulation on CT ( Figure 2). These findings, in conjunction with the absence of lytic lesions, were inconsistent with Langerhans cell histiocytosis and supported a diagnosis of ECD. Apart from the pathological bone aspect, there have been signs of accumulated radiotracer in both renal cavities. A subsequent renal ultrasound confirmed bilateral dilatated pyelocaliceal cavities likely due to the periureteral fibrotic compression. A JJ ureteral stent, was placed to relieve obstruction, resulting in stabilisation of renal function.

**Figure 3.**
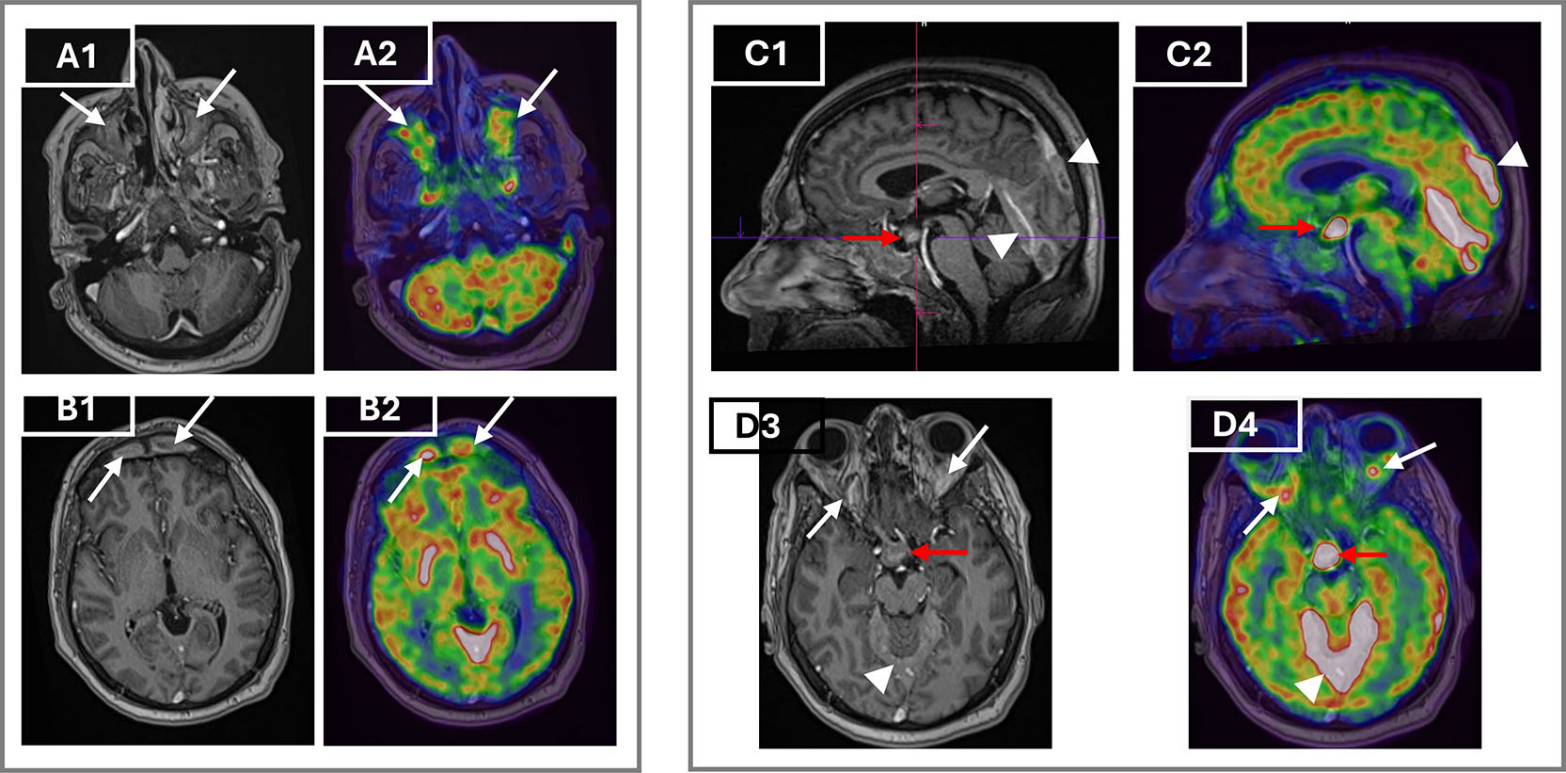
MRI and FDG PET/CT demonstrating orbital, craniofacial and central nervous system involvement in Erdheim–Chester disease before treatment. Axial post-contrast T1-weighted MRI (A1) and corresponding axial FDG PET/CT fusion image (A2) demonstrate intense enhancement and hypermetabolism of the maxillary sinuses (arrows). Axial post-contrast T1-weighted MRI (B1) and PET/CT (B2) show bilateral hypermetabolic frontal sinus involvement (arrows). Sagittal T1-weighted MRI (C1) and PET/CT (C2) reveal a suprasellar mass thickening the infundibulum (red arrows) and pachymeningeal enhancement of the tentorium cerebelli and adjacent meninges (arrowheads). Axial T1-weighted MRI (D3) and PET/CT (D4) show bilateral retroorbital masses (white arrows) encasing the optic nerves, along with hypermetabolism in the suprasellar mass (red arrows) and tentorium cerebelli (arrowheads). **Abbreviations: MRI** = magnetic resonance imaging; **FDG PET/C**T = fluorodeoxyglucose positron emission tomography/computed tomography.

**Figure 4.**
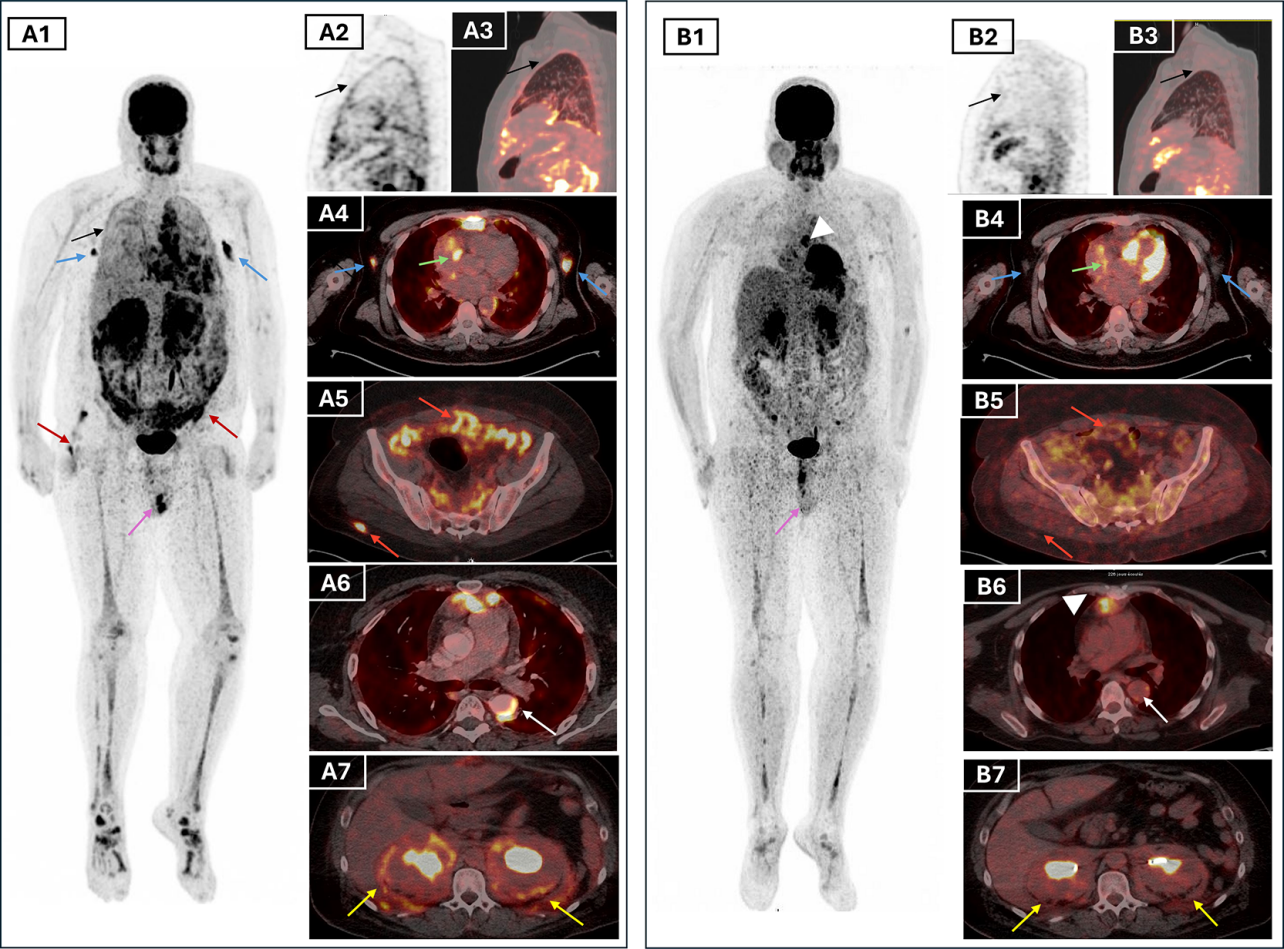
Pre-treatment FDG PET/CT (A) and post-treatment (B) MIP and axial fused PET/CT images illustrating multisystemic response in ECD. Lesions that resolved completely include pleural thickening (black arrows, A1–A3), axillary lymph nodes (blue arrows, A1), and testicular uptake (purple arrows, A1 and B1). Partial metabolic response was seen in periaortic vascular fibrosis (white arrows, A6 and B6), atrioventricular sulcus lesion (green arrows, A4 and B4), peritoneal and subcutaneous lesions (red arrows, A5–A6 and B5–B6), and perirenal/retroperitoneal “hairy kidney” involvement (yellow arrows, A7 and B7), with persistent uptake in the mediastinal retrosternal nodule (white arrowheads, B1 and B6). Bone uptake (A1, B1) remained visible but reduced *.* **Abbreviations**: **FDG PET/CT** = fluorodeoxyglucose positron emission tomography/computed tomography; **MIP** = maximum intensity projection; **ECD** = Erdheim–Chester disease.

To assess systemic disease burden and metabolic activity, a whole body 18F-FDG PET/CT was performed. The scan showed high standardised uptake values (SUV) with hypermetabolic retro-orbital masses (SUVmax=7.9), pachymeningeal thickening (SUVmax=28.3), and an active suprasellar lesion (SUV max=27.3), correlating with the patient’s known central diabetes insipidus and confirming hypothalamic-pituitary axis involvement ( Figure 3). Additionally, there was cardiac manifestations through intense uptake in retrosternal thickened tissue (SUVmax=10.2), pericardiac nodular thickening (SUVmax=12.1), perivascular fibrosis of the descending aorta and a focal hypermetabolism in the atrio-ventricular sulcus. There was also diffuse hypermetabolic pleural activity associated with mild bilateral effusion, periaortic bilateral axillary lymphadenopathy, peri-renal and retroperitoneum fibrosis (SUV max=7), epiploic fat nodules, bilateral testicular hypermetabolism (SUV max=9) and subcutaneous nodular lesions in the right gluteal region ( Figure 4A). The extent of the skeletal involvement was also confirmed through the hypermetabolic symmetric osteosclerotic lesions of particularly the long and feet bones and craniofacial bones (SUVmax=4.9) ( Figure 4A).

MRI of the brain and orbits complemented PET/CT by showing retrobulbar soft tissue thickening encasing the optic nerves, hypothalamic mass with infundibular thickening, and pansinusitis. These findings further supported CNS involvement ( Figure 3).

A transthoracic echocardiography showed concentric left ventricular hypertrophy with preserved ejection fraction (55-60%).

**Figure 5.**
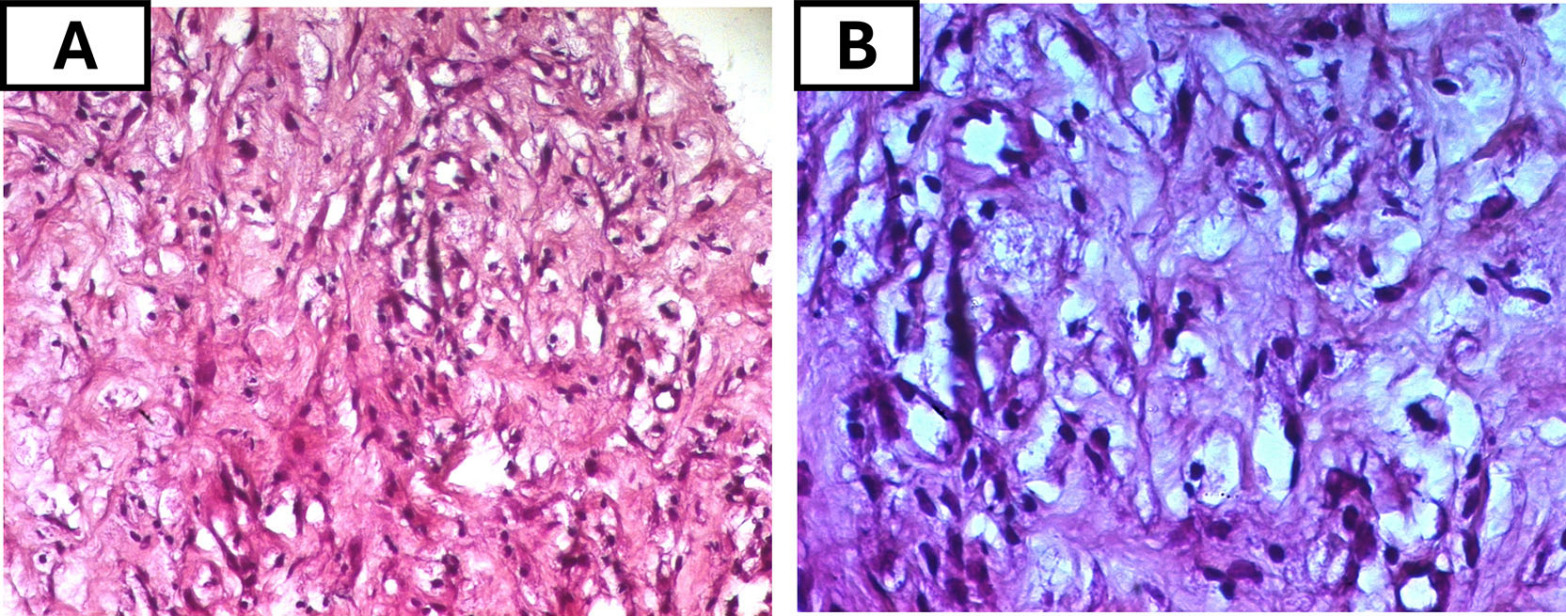
Histopathological features of ECD on hematoxylin and eosin staining. (A) Intermediate-power view (H&E, original magnification ×100) showing sheets of foamy histiocytes with abundant pale, vacuolated cytoplasm within a fibrotic background. (B) High-power view (H&E, ×200) highlighting the large lipid-laden histiocytes with small, eccentric nuclei *.* **Abbreviations**: **H&E** = hematoxylin and eosin; **ECD** = Erdheim–Chester disease

Initial bone biopsies of the left tibia and maxillary bones were inconclusive. Consequently, a CT guided biopsy of the retroperitoneal tissue was performed and showed the fibrous infiltration with CD68-positive, CD1-negative foamy histiocytes ( Figure 5), confirming a non-Langerhans histiocytosis compatible with ECD and further ruling out Langerhans cell histiocytosis. The patient also tested negative for the JAK2 mutation, effectively ruling out an associated myeloproliferative neoplasm, which can occasionally co-occur with ECD. Molecular testing for the BRAFV600E mutation and other MAPK pathway alterations was ordered but was not conducted due to financial restrictions, and thus the BRAF mutation status remained pending to date.

First line therapy included the administration of corticosteroids and methotrexate with a dose of 10 mg on day 1 and 25 mg on Day 2 weekly.

Given the multisystem disease, including cardiovascular, CNS and the unknown BRAF status, the patient was initiated on weekly subcutaneous pegylated interferon alpha at a dose of 180 micrograms. Clinical improvement was evident within weeks, with resolution of fever, improved general status, and normalisation of inflammatory markers (CRP declined from 229 to 14 mg/L). The ophthalmologic assessment revealed stable right exophthalmos without visual impairment. The patient tolerated the treatment well with no documented side effects. A follow-up 18F-FDG PET/CT scan after 7 months of therapy showed a partial metabolic response, with reduction in SUV values and size of the suprasellar lesion and pericardial thickening, complete regression of bilateral testicular and pleural hypermetabolism, and improvement of the axillary lymphadenopathy ( Figure 4B). Persistent, though attenuated, uptake was still noted in the long bones and paranasal sinuses, consistent with residual disease ( Figure 4B). These findings validated the early efficacy of interferon-α, and highlighted PET/CT as an essential modality for treatment monitoring in ECD ( Table 1).

**Table 1.** Chronological summary of clinical presentation, diagnostic investigations, and management in the reported case.

Date/Interval	Event
1 year prior to admission	**Diagnosis of central diabetes insipidus, with desmopressin therapy**
6 weeks prior to admission	**Bilateral ankle pain without swelling or erythema**
15 days prior to admission	**Onset of persistent fever and retrosternal chest pain**
Day 0-Day 3	**Hospital admission, infectious causes excluded; urgent CT angiography shows mediastineal fat infiltration and small percardial effusion, with a “hairy kidneys” appearance**
Day 4-Day 10	**Whole body 99mTc-HDP bone scintigraphy and SPECT/CT performed; typical findings of skeletal uptake in ECD**
Day 10-Day 14	**FDG PET/CT confirms multisystem involvement, including CNS, cardiac, retroperitoneal and skeletal lesions**
Day 14	**CT guided retroperitoneal biopsy confirms ECD**
Week 3	**Initiation of corticosteroids and pegylted interferon-alpha therapy**
Week 4 onwrads	**Cinical improvement, fever resolved, inflammation biochemical response**
Month 7 of treatment	**Follow- FDG PET/CT shows patial morphologic and metabolic response nd regression of several lesions**

**Abbreviations**: **CT **= computed tomography; **99mTc-HDP **= Technetium-99m hydroxymethylene diphosphonate; **SPECT =** single-photon emission computed tomography; **FDG PET **= fluorodeoxyglucose positron emission tomography; **CNS =** central nervous system; **ECD **= Erdheim–Chester disease.

## Discussion

ECD is a rare histiocytic disorder, with fewer than 2000 cases reported to date since its discovery in 1930. ^4^ The disease mostly affects male adults with a mean age at diagnosis at 55 and sex ration of 3:1. Childhood onset is exceptionnally rare with only 20 recorded cases in history. ^4^

Within the xanthogranuloma family, it has a spectrum of histopathological features and is characterized by the infiltration of tissues by foamy CD68, factor XIIIa, and negative for CD1a and CD207 (langerin) histoiocytes. ^7^ Rosai-Dorfman disease (RDD), Along with juvenile xanthogranuloma (JXG), ALK-positive histiocytosis (APH), and histiocytic sarcoma (HS), it belongs to the histiocytic neoplasms in the The 5th edition of the World Health Organization (WHO) Classification of Haematolymphoid Tumours. ^8^

The typical cause of the disease is constitutive MAPK signaling pathway mutations which are one of the first targets for molecular therapeutics for histiocytes. ^4, 7^ The disease is clinically heterogenous, varying according to the location and extent of organ involvement. Its natural course is characterized by the progressive accumulation of lesions in multiple systems with spontaneous regression being only rarely observed and are fatal if left untreated. ^7^

Diagnosis is often delayed due to variable clinical presentations. Endocrine dysfunction, particularly diabetes insipidus is observed in 25-30% of patients and may precede diagnosis by years and is a frequent early manifestation. ^9, 10^

While bone osteosclerosis occurs in more than 90% of cases, bone pain only appears in only half of patients usually localized in knees and ankles ^3, 4^ which was the case of our patient.

Extraskeletal involvement includes retroperitoneal fibrosis, pericardial and mediastinal infiltration as well as CNS lesions. ^11^

Our patient’s clinical course manifested the typical multisystemic pattern of ECD but also presented several distinctive features. Fever of unknown origine (FUO), and elevated inflammatory markers were dominant early features marking the atypical inflammatory presentation. Constitutional symptoms such as fatigue, night sweats, and weight loss are reported but not among the most frequent features, ^4, 11^ implying its relative rarity in ECD cases. This systemic feature is in line with the Th-1 mediated systemic immune activation described in ECD. Arnaud et al. ^12^ reported elevated IL-12, IFN-α, and CP-1, with increased IL-6 in untreated patients. These cytokines are implicated in both systemic inflammatory responses and bone remodeling. This disease specific profile likely underlies the atypical inflammatory onset in our case.

Accurate diagnosis and optimal treatment planning in CED relay heavily on multimodality imaging, including MRI, CT, and nuclear medicine hybrid imaging.

Symmetric retroperitoneal fibrosis and perirenal sheathing (“hairy kidneys”), which are hallmarks of ECD, were discovered by CT and are present in a third of patients. ^13^ Retroperitoneal fibrosis can also arise as an IgG4-related disorder or related to other causes, but in ECD it most often circumferentially encases the abdominal aorta and involves the perirenal space. This contrasts with IgG4-related disease, which typically affects the anterolateral aortic wall and displaces rather than obstructs the distal ureters. ^14^ Our patient had ureteral obstruction which required JJ stent placement. This intervention alleviated renal hydronephrosis, reflecting a known complication of ECD-related fibrosis. ^13^

Other CT findings included loss of the fat interface with the myocardium and dense, infiltrative thickening of the anterior mediastinal fat and pericardium as well as the infiltration of the atrio-ventricular sulcus and the peri-aortic infiltration. These changes are commonly observed in ECD cardiac involvement which are usually asymptomatic. ^5, 15^ Our patient, however, presented with chest pain, and one possible explanation could be coronary stenosis which is reported in 23% of cases. ^5^ A dedicated coronary angiography could be helpful in more accurately assessing the extent and severity of coronary involvement.

MRI of the brain explained the long-standing DI as it identified a suprasellar mass and pituitary stalk thickening, present in 24% of ECD patients as the first clinical manifestation, and usually several years before ECD diagnosis. ^4^ The importance of histologic confirmation is emphasized by the possibility that these results could be mistakenly attributed to Langerhans cell histiocytosis or other adenomatous, granulomatous or inflammatory processes of this region. ^11^ Notably, CNS involvement occurs in about half of ECD patients and is associated with increased mortality. ^16^ Clinical manifestations vary and may include DI, exophthalmos, cerebellar ataxia, cranial nerve palsies, and cognitive impairment, ^16^ paralleling the site and extent of radiologic lesions. Like in our patients, reports show that ECD lesions are often extra-axial, contiguous with facial or orbital bone, and may be associated with osteosclerosis of the clavarial and facial bones. ^17^ Dural and meningeal involvement, seen in up to 23% of cases, ^16^ can present as diffuse thickening or mass-like lesions. ^17^ The coexistence of facial bone osteosclerosis with orbital or meningeal disease, as in our patient, is a recognized association that may help direct clinicians toward the diagnosis of ECD. ^16^

SPECT/CT of the bone scintigraphy provided anatomical-functional correlation, showing strikingly tracer uptake corresponding to metaphyseal osteosclerosis of long bones and facial bones. Associated epiphyseal sparing ruled out other conditions responsible for radiotracer symmetric uptake like progressive diaphyseal dysplasia, Gaucher disease and multifocal osteonecrosis. ^13^ Notably, unlike Langerhans cell histiocytosis, the mandible and axial skeleton are typically spared. This modality proved particularly helpful for bone mapping and diagnosis confirmation with the typical uptake distribution.

FDG PET/CT was crucial in depicting the full extent of the disease, revealing both typical and atypical sites of extraosseous involvement. ^18^ It showed mirrored skeletal lesions to those in bone scintigraphy. It also confirmed hypermetabolic lesions in the CNS, right atrium, pericardium, pleura, retro-orbital regions, retroperitoneum, and bilateral axillary lymph nodes, while also identifying bilateral testicular involvement, a particularly rare manifestation of ECD. ^15^ Importantly, the scan also revealed hypermetabolic involvement of the omentum, peritoneum, and subcutaneous soft tissues in the form of hypermetabolic nodules. When reviewing patterns in ECD, Young et al. ^3^ proposed that higher SUV values may correlate with BRAF mutation status. PET/CT’s superior sensitivity compared to CT or bone scan was also illustrated by Kim et al., ^19^ where ECD lesions were occult on structural imaging but evident on FDG uptake. Follow-up PET/CT showed partial response to treatment, with phenomenal metabolic regression in most of the pathological sites. In our patient, follow-up PET/CT demonstrated only residual moderate perirenal hypermetabolism and less extensive bone manifestations along with the persistence of moderate to high FDG uptake in the nodular lesions of the retrosternal space and the pericardium.

The patient’s persistent thrombocytosis raised suspicion for an associated myeloproliferative neoplasm, as hematologic malignancies could be an overlap in up to 10% of ECD patients. ^1^ However, JAK2 mutation testing was negative.

While almost half of the patients (50-60%) harbor a BRAFV600E mutation ^15^ and its identification is essential not only for confirming diagnosis but also for directing mutation-specific therapy. However, false-negative results can occur because of mosaic distribution of the mutation and low variant allele frequency in some biopsy specimens. ^20^ Therefore, molecular testing should be pursued on **multiple tissue sites** (for example perirenal, bone or skin lesions) and with **high-sensitivity assays** to reduce the risk of false negatives and to allow timely access to BRAF or MEK inhibitors such as the **BRAF inhibitor vemurafenib** when indicated. ^7^^,^^20^ The presence of a BRAF mutation could also indicate an FDG avid CNS disease and associated with worse outcome as found by Young et al. ^3^

The BRAF status of our patient is still unknow, as the molecular analysis is still pending; however, the CNS lesions demonstrated disproportionately higher SUV values compared to other organ sites, suggesting greater metabolic activity in this region, which further supports the assumption that our patient has a BRAF-positive status. This limited therapeutic options to immunomodulators, primarily interferon-α (IFN-α) which remain the cornerstone of treatment and the currently conventional therapy with largest evidence-base in ECD. ^7^ Its preferable pegylated formulation is recommended and increasing doses may be needed in cases of CNS and cardiovascular manifestations ^7^ as was the case in our patient.

The rapid fever resolution and CRP normalization with IFN-α and Methotrexate align with prior reports of IFN-α efficacy in systemic inflammation. ^21^ This regimen achieved fever resolution within 72 hours of corticosteroid initiation, stabilization of cardiac and CNS lesions at 7-month follow-up. IFN-α is known the high prevalence of side effects that our patient didn’t present. They include fatigue, depression and cytopenia. ^7^ This treatment proved to be a crucial therapeutic option for the patient in a setting where targeted therapies are unavailable and the BRAF mutation status remained unknow to date.

In contrast to cases where response to IFN-α was poorest in the CNS, lungs, and heart, ^21^ our patient demonstrated a favorable partial response as shown on the 7-month follow-up PET/CT scan.

This case further illustrates the utility of PET/CT in monitoring treatment response and guiding follow-up. ^11^ That was particularly in osseous and soft tissue lesions that are nor evaluable by conventional anatomic criteria. This reflects growing evidence that PET-based metrics, such as modified PERSIST, may be better suited than RECIST for assessing response in ECD. ^18^ Although expert consensus supports FDG PET/CT for response assessment in ECD and other histiocytic disorders, the correlation between metabolic changes and clinical benefit still requires further validation in prospective studies. ^7, 18^

A sustained partial metabolic response in the setting of clinical improvement represents a favorable outcome, ^7^ that’s why, the patient was put under surveillance with the current treatment along with a planned additional PET/CT scan.

## Conclusion

This case illustrates the diagnostic and therapeutic challenges of Erdheim-Chester disease and highlights the critical role of multimodal imaging in guiding clinical decision-making. Our patient presented with fever of unknown origin preceding central diabetes insipidus and ultimately diagnosed of extensive multiorgan manifestations of ECD including rare ones. The unknown BRAF mutation status along with cardiac and CNS involvement in the setting of limited resources led to the pegylated IFN-α treatment. The obstructive hydronephrosis urged the need for urologic intervention. This case further reinforces current recommendations supporting FDG PET/CT as a valuable tool for monitoring disease response. Multidisciplinary collaboration remains essential in optimizing care for patients with rare, multisystem histiocytosis such as ECD.

### Strengths and limitations

The strengths of this report include the documentation of a rare inflammatory presentation with FUO as a dominant early sign, the use of comprehensive multimodal imaging to map both typical and atypical disease sites, and longitudinal PET/CT follow-up demonstrating partial metabolic response despite CNS and cardiac involvement.

Limitations include the single-case nature, pending molecular results, which restricted mutation-directed therapy selection and the short-term follow-up relative to the chronic disease course.

### Learning points

• ECD as an FUO Mimic: Persistent fever with endocrine abnormalities (e.g., DI) warrants ECD evaluation, even in younger patients.
• Imaging Triad: (FDG PET/CT and SPECT/CT (metabolic mapping), MRI (CNS/orbital detail), and CT (“hairy kidney”) are synergistic in ECD diagnosis.
• Multidisciplinary Care: Urologic intervention (stenting) is critical for obstructive complications.
• Extensive multisystem disease with CNS and cardiac involvement with an unknow BRAF-status: Pegylated IFN-α + methotrexate offers accessible, effective and a viable regimen for first-line therapy.

### Patient perspective

The patient reported having dealt with unexplained fever and pain for weeks without any clear explanation for the diabetes insipidus for almost a year. Following the initiation of treatment, most symptoms were resolved, and he expressed relief at finally receiving a definitive diagnosis.

## Ethical approval

Ethical approval was not required.

## Consent

Written informed consent for publication of their clinical details was obtained from the patient.

## Contributor information

Hajer Boudriga: hajer.boudrigua@famso.u-sousse.tn

Imen Ben Hassine: imenbenhassine1987@gmail.com

Omar Ben Elkilani: benelkilaniomar@gmail.com

Raja Sfar: sfarraja@yahoo.fr

Abir Ezzine: abirezzine.bi@gmail.com

Jihed Anoun: miledjihed@gmail.com

Anis Mzabi: mzabi_anis@yahoo.fr

Fatma Ben Fredj: bfi.fatma@yahoo.fr

Kaouthar Chatti: kaouthar.chatti@gmail.com

## Disclosure of AI use

The authors used ChatGPT (OpenAI, GPT-4, 2025 version) to assist in correcting minor language errors and improving the sentence formulation in the manuscript. All authors reviewed and approved the final manuscript to ensure its accuracy and integrity.

## Data Availability

No data are associated with this article. All relevant supporting materials, including the completed CARE checklist, are openly available in Zenodo. ^22^ This project contains the following extended data: “CARE Checklist for case report on “When an unknown fever unmasks Erdheim-Chester disease: a case showing the use of multimodality imaging and the successful interferon alpha therapy”. DOI: https://doi.org/10.5281/zenodo.16794958. ^22^ Data is available under the terms of the **Creative Commons Zero v1.0 Universal** license.
