# Supplementary material for: Case Report: When an unknown fever unmasks Erdheim-Chester disease: a case showing the use of multimodality imaging and the successful interferon alpha therapy
Source: F1000Res. 2025 Oct 17;14:838. Originally published 2025 Aug 29. [Version 2] doi: 10.12688/f1000research.169202.2 (PMC12589941; doi:10.12688/f1000research.169202.2)
Supplement: Peer review history

# Reviewer report: Reviewer response for version 2

**DOI:** 10.5256/f1000research.189785.r425046

**Published:** 2025-11-06

**Authors:** Antonio Todisco

**Affiliations:** 1 Neurology, Università Campus Bio-Medico di Roma, Rome, Italy

Corrections have been made.

Are enough details provided of any physical examination and diagnostic tests, treatment given and outcomes?

Yes

Is the case presented with sufficient detail to be useful for other practitioners?

Partly

Is sufficient discussion included of the importance of the findings and their relevance to future understanding of disease processes, diagnosis or treatment?

Yes

Is the background of the case’s history and progression described in sufficient detail?

Yes

Reviewer Expertise:

Neuroimmunology, neurodegenerative disease, rare neurological disease, neuromuscular disease, EMG

I confirm that I have read this submission and believe that I have an appropriate level of expertise to confirm that it is of an acceptable scientific standard.

# Reviewer report: Reviewer response for version 2

**DOI:** 10.5256/f1000research.189785.r425045

**Published:** 2025-10-28

**Authors:** Hatem Nasr

**Affiliations:** 1 Nuclear Medicine, Cairo University Kasr Alainy Faculty of Medicine, Cairo, Cairo Governorate, Egypt; 2 Cairo University, Giza, Egypt

I would like to thank the authors for the their efforts and amendments performed for this clinically valuable case report.

Are enough details provided of any physical examination and diagnostic tests, treatment given and outcomes?

Yes

Is the case presented with sufficient detail to be useful for other practitioners?

Yes

Is sufficient discussion included of the importance of the findings and their relevance to future understanding of disease processes, diagnosis or treatment?

Yes

Is the background of the case’s history and progression described in sufficient detail?

Yes

Reviewer Expertise:

Nuclear Medicine, nuclear oncology, nuclear cardiology, radionuclide brain imaging, PET/CT imaging, SPECT/CT imaging, theranostics, infection imaging, radionuclide imaging, transarterial radionuclide embolization (TARE), Ga-68 DOTATATE imaging, F-18-FES imaging, PSMA imaging, F-18 DOPA imaging

I confirm that I have read this submission and believe that I have an appropriate level of expertise to confirm that it is of an acceptable scientific standard.

# Reviewer report: Reviewer response for version 1

**DOI:** 10.5256/f1000research.186495.r417076

**Published:** 2025-10-14

**Authors:** Hatem Nasr

**Affiliations:** 1 Nuclear Medicine, Cairo University Kasr Alainy Faculty of Medicine, Cairo, Cairo Governorate, Egypt; 2 Cairo University, Giza, Egypt

Overall Evaluation

 This case report provides a valuable contribution to the literature on *Erdheim–Chester Disease (ECD)*, a rare histiocytic neoplasm. It demonstrates a well-illustrated multimodal diagnostic pathway and documents clinical improvement with pegylated interferon-α.

 The report offers an educational value to clinicians managing rare multisystemic diseases.

**1. Title and Abstract**

-
  - The title is informative, concise, and effectively summarizes the case’s novelty (fever as a presenting symptom, multimodality imaging, interferon therapy).
  - The abstract is well-structured, outlining the clinical background, case details, and main outcomes.

**2. Introduction**

- **Strengths:**
  - Provides a succinct and accurate overview of ECD, with up-to-date references.
  - Highlights the diagnostic complexity and role of imaging.
- **Limitations:**
  - In the 2 ^nd^ paragraph the word “hypopituitarism” is mistakenly repeated.

**3. Case Presentation**

- **Strengths:**
  - Exceptionally detailed chronology, thorough clinical, imaging, and histologic data.
  - Use of multiple imaging modalities (CT, SPECT/CT, MRI, PET/CT) enhances diagnostic rigor.
  - Inclusion of clear, well-annotated figures.
- **Limitations:**
  - No quantitative PET/CT metrics (SUV values).
  - The *BRAF* mutation testing being unavailable weakens the precision of molecular diagnosis.
  - Lack of follow-up beyond 7 months limits understanding of long-term treatment efficacy.

**4. Discussion**

- **Strengths:**
  - Balanced incorporation of clinical, radiologic, and therapeutic aspects supported by contemporary literature.
  - Explains atypical presentation (fever of unknown origin) with plausible immunologic mechanisms.
  - Discusses differential diagnoses and imaging correlations comprehensively.
- **Limitations:**
  - Some repetition across imaging discussions could be condensed for clarity.

**5. Conclusion**

-
  - Clear and concise; emphasizes multimodal imaging and multidisciplinary care.

**6. References**

- **Strengths:**
  - Recent and high-quality sources.
  - Properly formatted with DOIs and PubMed links.
- **Limitations:**
  - Duplication (entries 16 and 18 both cite Papo et al., 2019).
  - One reference (“Wrong Erdheim-Chester Disease”) appears mis-titled.

**Final Opinion:**

 The case report is scientifically sound and contributes meaningful insights into a rare disease diagnostics and management.

 This is a well-prepared and clinically valuable case report that merits indexing following minor revisions.

**Final Recommendation**

**Decision: Accept with minor revisions**

Are enough details provided of any physical examination and diagnostic tests, treatment given and outcomes?

Yes

Is the case presented with sufficient detail to be useful for other practitioners?

Yes

Is sufficient discussion included of the importance of the findings and their relevance to future understanding of disease processes, diagnosis or treatment?

Yes

Is the background of the case’s history and progression described in sufficient detail?

Yes

Reviewer Expertise:

Nuclear Medicine, nuclear oncology, nuclear cardiology, PET/CT imaging, SPECT/CT imaging, theranostics, infection imaging, radionuclide imaging, transarterial radionuclide embolization (TARE), Ga-68 DOTATATE imaging, F-18-FES imaging, PSMA imaging, F-18 DOPA imaging

I confirm that I have read this submission and believe that I have an appropriate level of expertise to confirm that it is of an acceptable scientific standard.

# Response

**Published:** 2025-10-15

**Authors:** Hajer Boudriga

**Affiliations:** Department of Nuclear Medicine, University of Sousse, Sousse, Sousse, Tunisia

We sincerely thank the reviewer for their thorough and constructive evaluation of our manuscript entitled

 We appreciate the positive comments regarding the educational value, clarity of presentation, and comprehensive multimodal diagnostic approach. Below, we address each point raised.

**Title and Abstract:**

 We thank the reviewer for this positive assessment. No further changes were required in this section.

**Introduction:**

 We appreciate the reviewer’s attention to detail. The redundant occurrence of the word *“hypopituitarism”* in the second paragraph has been corrected in the revised version.

**Case Presentation:**

- We have now included **quantitative PET/CT data (SUVmax values)** in the *Case Presentation* and highlighted that **the CNS lesions demonstrated disproportionately higher SUV values** compared to other organ sites.
- The issue of unavailable **BRAF mutation testing** has been addressed and expanded in the *Discussion*. We added a paragraph explaining the **diagnostic and therapeutic importance of BRAF testing**, the **risk of false negatives due to mosaic mutation distribution**, and the recommendation for **multi-tissue sampling and high-sensitivity methods**
- Regarding **follow-up**, we have acknowledged this as a study limitation in the *Strengths and Limitations* section, clarifying that follow-up remains ongoing.

**Conclusion:**

 We thank the reviewer for this positive feedback.

**References:**

 Both issues have been corrected. Duplicate references were removed, and the incorrect title has been replaced with the accurate citation. All references were rechecked for formatting consistency.

 Finally, we are grateful for the reviewer’s insightful remarks, which have strengthened the clarity, accuracy, and scientific rigor of our case report.

# Reviewer report: Reviewer response for version 1

**DOI:** 10.5256/f1000research.186495.r410847

**Published:** 2025-10-09

**Authors:** Antonio Todisco

**Affiliations:** 1 Neurology, Università Campus Bio-Medico di Roma, Rome, Italy

Boudriga and colleagues presented an interesting case report, namely: "When an unknown fever unmasks Erdheim-Chester disease: a case showing the use of multimodality imaging and the successful interferon alpha therapy". They comprehensively describe the clinical history and therapeutic management, in particular in a low-resource setting in absence of BRAF characterization.

 The discussion is adequate and the diagnostic approach is clear. However, since the BRAF mutation was not characterized, that part of the diagnostic/therapeutic approach is not complete. In case of ECD, the role of BRAF analysis is crucial to start a specific molecular targeted therapy and, sometimes, multiple testing is necessary, due to false negatives. I suggest to integrate few words about it, as described in Todisco et al [Reference 1].

 The English is good and fluent.

Are enough details provided of any physical examination and diagnostic tests, treatment given and outcomes?

Yes

Is the case presented with sufficient detail to be useful for other practitioners?

Partly

Is sufficient discussion included of the importance of the findings and their relevance to future understanding of disease processes, diagnosis or treatment?

Yes

Is the background of the case’s history and progression described in sufficient detail?

Yes

Reviewer Expertise:

Neuroimmunology, neurodegenerative disease, rare neurological disease, neuromuscular disease, EMG

I confirm that I have read this submission and believe that I have an appropriate level of expertise to confirm that it is of an acceptable scientific standard, however I have significant reservations, as outlined above.

## References

[1] : Erdheim-Chester disease: A challenging diagnosis for an effective therapy. *Clinical Neurology and Neurosurgery* .2020;194: 10.1016/j.clineuro.2020.105841 10.1016/j.clineuro.2020.105841 32330799

# Response

**Published:** 2025-10-15

**Authors:** Hajer Boudriga

**Affiliations:** Department of Nuclear Medicine, University of Sousse, Sousse, Sousse, Tunisia

We thank the reviewer for this valuable comment emphasizing the importance of **BRAF mutation analysis** in the diagnostic and therapeutic management of ECD.

 We have now expanded the *Discussion* section to address this point. Specifically, we added a paragraph highlighting that:

- Molecular testing for **BRAFV600E** is a key diagnostic and therapeutic step in ECD.
- **False-negative results** may occur due to mosaic distribution or low variant allele frequency, as described by Todisco et al.
- Performing molecular analysis on **multiple tissue specimens** using **high-sensitivity techniques** is recommended to confirm mutation status and guide targeted treatment.
- In our case, the absence of local molecular testing constrained the therapeutic strategy to interferon-α–based therapy, which remains an effective alternative in resource-limited settings.
